# Molecular Dynamics Simulation Reveals Exposed Residues in the Ligand-Binding Domain of the Low-Density Lipoprotein Receptor that Interacts with Vesicular Stomatitis Virus-G Envelope

**DOI:** 10.3390/v11111063

**Published:** 2019-11-15

**Authors:** Faisal A. Al-Allaf, Zainularifeen Abduljaleel, Mohiuddin M. Taher, Ahmed A. H. Abdellatif, Mohammad Athar, Neda M. Bogari, Mohammed N. Al-Ahdal, Futwan Al-Mohanna, Zuhair N. Al-Hassnan, Kamal H. Y. Alzabeedi, Talib M. Banssir, Abdellatif Bouazzaoui

**Affiliations:** 1 Department of Medical Genetics, Faculty of Medicine, Umm Al-Qura University, P.O. Box 715, Makkah 21955, Saudi Arabia; zainulbio@gmail.com (Z.A.); taher23223@yahoo.com (M.M.T.); athar80@gmail.com (M.A.); nmbogari@uqu.edu.sa (N.M.B.); 2 Science and Technology Unit, Umm Al Qura University, P.O. Box 715, Makkah 21955, Saudi Arabia; 3 Molecular Diagnostics Unit, Department of Laboratory and Blood Bank, King Abdullah Medical City, Makkah 21955, Saudi Arabia; 4 Pharmaceutics Department, College of Pharmacy, Qassim University, Qassim, 51452, Saudi Arabia; a.abdellatif@qu.edu.sa; 5 Pharmaceutics and Industrial Pharmacy, Faculty of Pharmacy, Al-Azhar University, Assiut 71524, Egypt; 6 Department of Medical Genetics, King Faisal Specialist Hospital and Research Center, P.O. Box, 3354 Riyadh 11211, Saudi Arabia; ahdal@kfshrc.edu.sa (M.N.A.-A.); futwan@kfshrc.edu.sa (F.A.-M.);; 7 Regional Laboratory, Makkah 25321, Saudi Arabia

**Keywords:** familial hypercholesterolemia, coronary artery disease, sudden cardiac death, low-density lipoprotein receptor (LDLR), lentiviral vector system, fusion protein, transfection, transduction, I-TASSER, Molecular Operating Environment, molecular dynamics simulation, MOPAC2009, CHARMM, Gromacs, pyDock

## Abstract

Familial hypercholesterolemia (FH) is an autosomal dominant disease most often caused by mutations in the low-density lipoprotein receptor (LDLR) gene, which consists of 18 exons spanning 45 kb and codes for a precursor protein of 860 amino acids. Mutations in the LDLR gene lead to a reduced hepatic clearance of LDL as well as a high risk of coronary artery disease (CAD) and sudden cardiac death (SCD). Recently, LDLR transgenes have generated interest as potential therapeutic agents. However, LDLR packaging using a lentiviral vector (LVV) system pseudotyped with a vesicular stomatitis virus (VSV)-G envelope is not efficient. In this study, we modified the LVV system to improve transduction efficiency and investigated the LDLR regions responsible for transduction inhibition. Transduction efficiency of 293T cells with a 5′-LDLReGFP-3′ fusion construct was only 1.55% compared to 42.32% for the eGFP construct. Moreover, co-expression of LDLR affected eGFP packaging. To determine the specific region of the LDLR protein responsible for packaging inhibition, we designed constructs with mutations or sequential deletions at the 3′ and 5′ ends of LDLR cDNA. All constructs except one without the ligand-binding domain (LBD) (pWoLBD–eGFP) resulted in low transduction efficiency, despite successful packaging of viral RNA in the VSV envelope, as confirmed through RT-PCR. When we evaluated a direct interaction between LDLR and the VSV envelope glycoprotein using MD simulation and protein–protein interactions, we uncovered Val119, Thr120, Thr67, and Thr118 as exposed residues in the LDLR receptor that interact with the VSV protein. Together, our results suggest that the LBD of LDLR interacts with the VSV-G protein during viral packaging, which significantly reduces transduction efficiency.

## 1. Introduction

Familial hypercholesterolemia (FH) is a life-threatening autosomal co-dominant disease with a population prevalence of approximately 1 in 160,000–300,000 [1,2]. In approximately 90% of patients with FH, the disease results from mutations in the low-density lipoprotein receptor (LDLR), which is responsible for the elimination of LDL-cholesterol (LDL-C) from the blood by endocytosis and intracellular degradation [3]. Consequently, defects in the LDLR result in a partial or complete loss of LDLR function, leading to high levels of LDL-C in the serum, often with concentrations above 500 mg/dL. The accumulation of LDL-C to high levels results in the development of cardiovascular disease (CVD), and aortic valve and coronary artery disease in particular. Other genes that may affect LDL-C transport include apolipoprotein B (APOB), located in chromosome 2 (p24), and convertase subtilisin/Kexin type 9 (PCSK9) located in chromosome 1 (1p32.3) [1,4]. Mutations in APOB reduce the affinity of the LDLR, whereas gain-of-function mutations in PCSK9 cause high levels of LDLR degradation, because this gene is thought to be involved in the degradation of lysosomal LDLR protein [5]. This degradation results in reduced levels of receptor on the cell surface, and thus, to higher accumulation of LDL-C.

Treatment of FH, especially for homozygous individuals, remains challenging. Currently, the most effective therapeutic agents are 3-hydroxy-3-methylglutaryl-coenzyme A (HMG-CoA) reductase inhibitors, commercially known as statins [6]. Other drugs used to reduce LDL-C levels include recently approved mipomersen [7], lomitapide [8], evolocumab [9], niacin, and the cholesterol absorption inhibitor known as ezetimibe.

Ezetimibe can reduce LDL-C levels by approximately 10%–15% [10] with no side effects or liver toxicity [11,12]. To improve LDL-C levels, combining statin with niacin or ezetimibe is recommended and has an acceptable safety profile [13]. However, even after combination therapy, the majority of patients with homozygous FH will still maintain high LDL-C levels [10], and therefore, are at high risk for CVD. An aggressive program of plasma apheresis is also one of the most preferred treatments. However, the effect of such a regimen is transient and is not available to all patients [14]. Because approximately 75% of the total body LDL receptors are located in the liver, this organ is crucial for LDL metabolism [15]. Liver transplantation is, therefore, an efficient method for correcting LDL-C levels in most cases of homozygous FH [15,16,17,18,19], although risks associated with transplantation, long-term immunosuppression, and high morbidity and mortality limit the use of this approach. As an alternative, the delivery of functional LDLR transgenes to the liver has recently emerged as a promising therapeutic option for FH.

In the early nineties, Chowdhury et al. conducted ex vivo gene therapy in rabbits with LDLR defects and demonstrated a long-term improvement of hypercholesterolemia. Besides, they showed that animals receiving LDLR-transduced autologous hepatocytes had a 30%–50% decrease in the total serum cholesterol levels that persisted until the end of their experiment [20]. Grossman et al. later used a similar strategy to demonstrate the first gene therapy in subjects with homozygous FH [21]. However, they were not able to achieve many transduced hepatocytes and only caused a small reduction in LDL-C levels for three subjects. Kassim et al. then designed a recombinant adeno-associated vector 8 (AAV8) containing a mouse LDLR transgene under the control of a liver-specific thyroxine-binding globulin (TBG) promoter in a murine model. They achieved a significant reduction in total cholesterol levels within seven days, despite a hepatocyte transduction efficiency of only 5%–10% [22]. In a follow-up study from the same group, the authors did not report abnormal lipid accumulation in the livers or significant liver histopathology and T cell responses to the capsid and transgene [23].

Although further studies have attempted the use of different vectors and transgene constructs, none have shown stable expression with durable effects [24,25]. Furthermore, AAV has been shown to less efficiently transduce human hepatocytes than murine hepatocytes [26,27]. In this context, early preclinical and clinical studies [28,29] showed that rAAV2 vectors transduce human and mouse hepatocytes similarly, but at low levels. However, rAAV8 vectors transduce human hepatocytes approximately 20-fold less efficiently than they transduce mouse hepatocytes. Taken together, these studies demonstrate the feasibility of LDLR gene therapy, although there are concerns related to the development of severe side effects [23], including the destruction of hepatocytes by cell-mediated immunity [29] and significant differences between models [20,21,26,27]. These studies also revealed that preclinical animal models are not always predictive of results in humans [28,29,30]. For this reason, further studies are needed to achieve clinical use of LDLR gene therapy.

As mentioned in a previous study [27], the AAV capsid used for gene therapy significantly affects the transduction efficiency. In this study, we used a lentiviral vector (LVV) in combination with the vesicular stomatitis virus (VSV) envelope for the packaging of the LDLR transgene. Early studies have shown that VSV pseudotyped viruses exhibit broad tropism, high stability, and excellent transduction efficiency, making them the gold standard for gene transfer strategies [31,32]. The VSV-G envelop is robust and exhibits a high infectivity. The VSV tropism is remarkably broad and it is suggested that it enters the target cells via a wide-spread but undefined receptor. In the early nineties, Fischer et al. found an extracellular soluble protein that inhibits VSV infection [33], further protein sequencing, and immunoaffinity assays showed high homology of the soluble protein with human LDLR, and it was subsequently named soluble LDLR (sLDLR). Later, the same group found that the LDL receptor and its family members serve as receptors for VSV [34]. In this study, the authors have confirmed that sLDLR binds VSV and inhibits both infection by VSV and the transduction by a VSV-G-pseudotyped lentiviral vector. However, in a control experiment, the lymphocytic choriomeningitis virus (LCMV)-pseudotyped lentiviral vector (LV), the sLDLR did not bind to the LCMV-LV.

Recently, our group found that LDLR affects the packaging of eGFP. Use of an LVV pseudotyped with a VSV envelope and co-transfection of hLDLR with the eGFP plasmid in addition to the other packaging plasmids, led to the inhibition of the eGFP packaging, in a dose-dependent manner. This was confirmed in a publication from Otaha et al. [35]. It was shown that the inhibition of eGFP packaging could be a multifactorial process, interfering with different protein–protein interactions. The aims of this work is to study which parts of the LDLR reduced the transduction efficiency when we use the LDLR lentiviral vector system, pseudotyped with the VSV-G envelop, indeed different deletions in the LDLR sequence were introduced. Furthermore, we determine using the MD simulation, the residues that interact with the VSV protein.

## 2. Material and Methods

### 2.1. Construction of a 5′-LDLReGFP-3′ Fusion Cassette and Packaging of a Lentiviral Vector (LVV)

The 5′-LDLReGFP-3′ fusion cassette is under the control of the cytomegalovirus promoter (pCMV). The predicted sequence from 5′ to 3′ includes a multiple cloning site (MCS), a Kozak consensus sequence (GCC GCC ACC), hLDLR cDNA with the stop codon deleted (but inclusive of the signal peptide, LDL-binding domain, EGF homology domain, *O*-linked sugar domain, membrane anchor, and the cytoplasmic domains), a 21 bp spacer sequence (GGT CTA GAA CCG GTC GCC ACC), eGFP cDNA, and a MCS at the 3′ end (Supplementary Figure S1). The 21-nucleotide spacer between the LDLR and eGFP sequences codes for glycine, leucine, glutamic acid, proline, valine, alanine, and threonine. The proline residue was incorporated intentionally in the spacer to enhance its flexibility. Proline residues are known to prevent the formation of secondary structures and consequently, protein misfolding in many peptides [36,37,38]. The fusion cassette was synthesized by ATG Biosynthetic (Merzhausen, Germany) and cloned into the vector pLVX-IRES-ZsGreen between the 5’ LTR and 3’LTR. Packaging was performed using two auxiliary plasmids [31], one coding for the viral proteins group-specific antigen (gag), polymerase (pol), trans-activator of transcription (Tat), and Rev protein (Rev) (pCMV-∆R8.9), and the other coding for the VSV envelope protein (pMD2). Localization of the LDLR therapeutic gene and the eGFP reporter gene on a single vector was intended to complement LDLR deficiency and enable detection of the biodistribution pattern after vector-mediated gene transfer.

### 2.2. Transfection of 293T Cells Using Lipofectamine

Transfection and transient expression in 293T cells were performed as follows: a total of 1 × 10^6^ 293T cells were seeded in 24-well plates 24 h prior to transfection in Dulbecco’s modified Eagle medium (DMEM) supplemented with 10% FCS and 1% penicillin/streptomycin, and the culture medium was changed 2 h prior to transfection. A volume of 400 µL per well of DMEM without FCS and 1% penicillin/streptomycin was added to 24-well plates and incubated under 5% CO_2_ conditions. Next, a total of 0.25 µg of plasmid DNA (0.1 µg CMV∆8.91, 0.05 µg pMD2, and 0.1 µg M107 (for eGFP alone) or pLDLR–Egfp) in a total volume of 50 µL DMEM medium without FCS was mixed with 4 µL Lipofectamine from Invitrogen (Dreieich, Germany). The DNA/Lipofectamine mixture was incubated at 25 °C for 20 min, and 100 µL of the mixture was then added dropwise to cells. One hour later, 1 mL DMEM supplemented with 10% FCS and 1% penicillin/streptomycin was added. The medium was replaced one day later (14–16 h), and 48 h after transfection, the cells were observed using an Olympus IX71 fluorescence microscope (Hamburg, Germany) and FACS analysis was performed using a Cytomics FC500 Flow Cytometer from Beckman Coulter (Krefeld, Germany). The results were analyzed using WinMDI software and transfection activity was expressed as a percentage of positive cells.

### 2.3. Packaging and Transduction of 293T/HepG2 Cells with Supernatant

A total of 1 × 10^8^ 293T cells were seeded in 10 cm-diameter dishes 24 h before transfection in supplemented DMEM, and the culture medium was changed 2 h before transfection. A total of 2.5 μg of plasmid DNA (1 μg pLDLR–eGFP or M107, 0.5 μg of the pMD2 envelope plasmid, and 1 μg of packaging plasmid pCMV-ΔR8.9) was added to a 500 µL total volume of DMEM without FCS. The precipitate was formed by adding plasmid DNA to 40 µL Lipofectamine in a total volume of 500 µL DMEM medium without FCS and incubating at 25 °C for 20 min. Next, 1000 µL of the DNA/Lipofectamine mixture was added dropwise to cells. One hour later, 5 mL DMEM supplemented with FCS and 1% penicillin/streptomycin was added. The medium was replaced the next day, and after another 24 and 48 h, supernatant with the packaged virus was collected and filtered through 0.22 µm cellulose acetate filters. An equivalent amount of freshly harvested supernatant containing a packaged virus was used to transduce 1 × 10^5^ 293T cells (or HepG2 cells for constructs with a liver-specific promoter) in a 24-well plate. Two days post-transduction, cells were analyzed using an Olympus IX71 fluorescence microscope (Hamburg, Germany), and the transduction efficiency was quantified using FACS analysis with a Cytomics FC500 Flow Cytometer (Beckman Coulter). The results were analyzed using WinMDI software, and the transfection activity was expressed as a percentage of positive cells.

### 2.4. PCR using RNA from Viral Supernatant

RNA was isolated from viral supernatant using the QIAamp MinElute Virus Spin Kit from Qiagen (Hilden, Germany), and first-strand cDNA was synthesized using 20 ng of RNA (DNase-treated) in a 20 µL reaction mixture with the GoScript Reverse Transcription System from Promega (Mannheim, Germany), according to the manufacturer’s protocol. The GFP fragment was then obtained using 10 µL of cDNA as a template in a 20 µL total reaction mixture containing 0.4 µL Qiagen HotStarTaq plus DNA Polymerase (Hilden, Germany), 2 µL 10× PCR buffer, 2 µL 25 mM MgCl_2_, 0.4 µL 10 mM dNTPs, 2 µL of 10 µM forward/reverse primers (forward primer ATCGAGCTGAATGGCGATGT, reverse primer GATGTAGCCCTCAGGCATGG), and water. The PCR program consisted of polymerase activation at 95 °C for 5 min, followed by 40 cycles of denaturing at 95 °C for 30 s, annealing at 60 °C for 30 s, extension at 68 °C for 1 min, and a final extension at 68 °C for 5 min. The amplified product was separated on a 1.5% agarose gel to ascertain its size and quality.

### 2.5. Target Sequence, Template Identification, and Sequence Alignment

Because of the importance of overcoming the vector production problem, we examined which part of the LDLR domain could be responsible for failed lentivector production. We have addressed this problem using a bioinformatics approach, as described below.

The amino acid sequence of the conserved protein domain of lentivirus was obtained from the National Center for Biotechnology Information (NCBI) database (https://blast.ncbi.nlm.nih.gov/Blast.cgi). For template identification, the Basic Local Alignment Search Tool (BLAST) was used, and two BLAST methods, protein–protein BLAST and position-specific iterated-BLAST (PSI-BLAST), were performed for template selection by searching against the Protein Data Bank (PDB). After comparative searching, the homolog structure with the best score was selected as a template. The template protein PDB file and amino acid sequence data in FASTA format were downloaded from the PDB. Multiple sequence alignment of template and target proteins was performed using CLCbio software (Aarhus, Denmark). Alignments were then used to build a phylogenetic tree using the same software.

### 2.6. Protein Structure Modeling and Binding Site Prediction

Template-based protein structure determination was performed based on the following. Homologous proteins often have analogous tertiary structures. Consequently, the structure of a protein homolog of the targeted region is used to predict its structure. Also, conservation of the tertiary structure is much greater than that of the primary sequence. Interactive Threading ASSEmly Refinement (I-TASSER, University of Michigans, Ann Arbor, USA) constructs three-dimensional (3D) structural models by reassembling fragments excised from threading templates, starting with the amino acid sequence of the lentivirus target region [39]. The accuracy of these methods depends on the quality of templates and alignments. This method relies only on the tertiary coordinates of a target lentivirus domain template through the use of a large set of template model decoys to reconstruct the structure of the lentivirus target domain. Furthermore, we predicted the active site using the Molecular Operating Environment (MOE, Chemical Computing Group software, Montreal, Canada) [40]. An alpha-shape algorithm was used to determine potential active sites in the 3D protein model structures. To analyze the receptor active site, protein surface calculations and molecular docking were used to search for favorable binding configurations between a flexible protein and protein targets that are most likely to contribute to tight protein–protein interaction [41,42,43]. Typically, scoring functions emphasize favorable hydrophobic, ionic, and hydrogen bond contacts. We detected candidate protein–protein binding sites using an efficient geometric algorithm based on Edelsbrunner’s alpha-shapes. The LDLR versus the conserved domain of the lentivirus-binding site on a macromolecular structure was ranked according to its accessible hydrophobic contact surface, and active site analysis was used to identify polar, hydrophobic, acidic, and basic residues. Solvent-exposed ligand atoms, residues in close contact with the lentivirus domain atoms, and side-chain and backbone acceptors were visualized.

### 2.7. Molecular Docking and Molecular Dynamics (MD) Simulation

The LDLR protein structure selected with PDB ID: 1N7D was docked into the targeted active site of the group-specific antigen (gag) conserved domain determined by the docking server (http://www.dockingserver.com/web), which is based on AutoDock version 4 (Molecular Graphics Laboratory, San Diego, USA). The analysis evaluated accurate gag target domain geometry optimization, energy minimization, charge calculation, and docking calculation and provided an LDLR–gag conserved protein domain complex representation. We found that a more accurate partial charge calculation and therefore, a more accurate docking calculation, could be achieved using quantum chemical methods. For protein-to-protein docking calculations, quantum chemical partial charge calculations were used only for the target gag protein conserved domain. The application Molecular Orbital PACkage (MOPAC 2009, CAChe Research LLC, Portland, USA), which quickly calculates the partial charge of proteins by quantum mechanical semi-empirical methods, was also used. In addition, the targeted module is expected to assist advanced Molecular Dynamics (MD) simulation by Chemistry at Harvard Macromolecular Mechanics (CHARMM, Harvard University, Cambridge, USA) and Groningen Machine for Chemical Simulations (Gromacs, University of Groningen, Groningen, Netherland) and lead to an improved understanding of the structure and dynamics of complex biomolecular systems.

We also studied the interaction between the LDLR LBD (PDB ID: 1n7d) and VSV glycoprotein G ectodomain (PDB ID: 5i2s). This interaction was determined using pyDock (Barcelona, Spain), a protein–protein docking algorithm [44] based on electrostatics, desolvation energy, and a limited van der Waals interaction, to produce score rigid-body docking poses (pyDock SER) [45].

### 2.8. Constructs with Mutations and Sequential Deletions at 3′ and 5′ Ends of the LDLR cDNA or Liver-Specific Promoter

Using bioinformatics methods, we constructed a vector (construct A = pLDLRmut–eGFP) containing 7 mutations: mutation 1 (p38K > V), AAA (lysine) to GTT (valine); mutation 2 (p58E > K), GAG (glutamic acid) to AAA (lysine); mutation 3 (p72D>G), GAC (aspartic acid) to GGT (glycine); mutation 4 (p81R > D), CGC (arginine) to GAC (aspartic acid); mutation 5 (p572S > P), AGT (serine) to CCT (proline); mutation 6 (p648S > L), TCC (serine) to CTT (leucine); and mutation 7 (p688N > L), AAC (asparagine) to CTT (leucine) (Supplementary Figure S2C). We also designed other constructs (Supplementary Figure S4) with sequential deletions at the 3′ of LDLR cDNA of the 5′-LDLReGFP-3′ cassettes (construct B = pAD-eGFP, construct C = pOLS-eGFP, construct D = pEgf-eGFP, and construct E = pLBD-eGFP). We also produced two mutants of the 5′-LDLReGFP-3′ fusion cassettes with sequential deletions in the 5′ of the hLDLR cDNA sequence: the first corresponded to a deletion of the signal peptide (construct F = pWoSPep-eGFP) and the second to a deletion of the LBD (construct G = pWoLBD-eGFP). We also designed and constructed two additional vectors containing the same 5′-LDLReGFP-3′ fusion cassette but under the control of two different liver-specific promoters, albumin and Ealb-Pa1AT.

### 2.9. Data Availability Statement

All data generated and/or analyzed during this research study are available from the corresponding author on reasonable request.

## 3. Results

### 3.1. Transduction of 293T Cells with eGFP or pLDLR–eGFP

**Figure 1:**
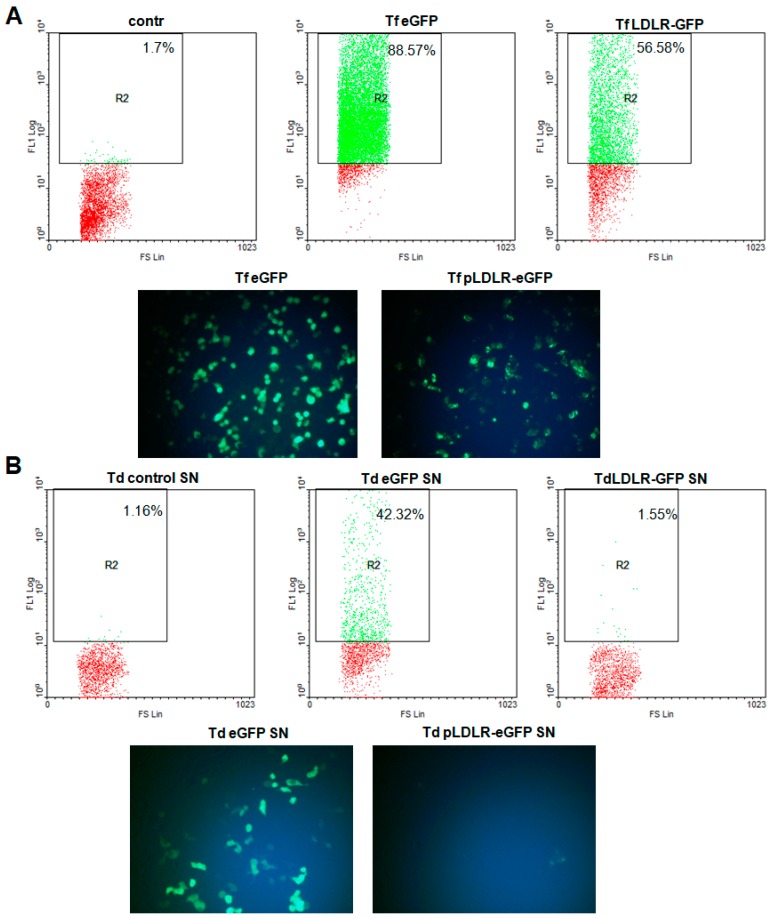
Transfection and transduction of 293T cells using LDLReGFP or eGFP. After transfection of the 293T cells in 24-well plates as described in the materials and methods section, (**A**) we analyzed the Expression of GFP in 293T cells transfected with construct harboring eGFP gene only or 5’-LDLReGFP-3’ cassette using fluorescent microscopy (original magnification, ×200) or flow cytometry and express the transfection efficiency in percent of positive cells. (**B**) After packaging of the construct harboring eGFP gene only or 5’-LDLReGFP-3’ cassette, as described in the materials and methods section, we used the same supernatant amount to transduce the 293T cells. Thereafter, cells were used for fluorescent microscopy (original magnification, ×200) and FACS analysis. The results were analyzed using WinMDI software and transfection efficiency was expressed in percent of positive cells.

Previous studies have identified the VSV-G-pseudotyped LVV as the gold standard for gene transfer strategies. For this reason, we used an LVV in combination with the VSV envelope (Supplementary Figure S1) to determine whether this combination can improve the transduction efficiency of the LDLR gene. After transfection and transduction, the efficiency was quantified using FACS and fluorescence microscopy. As shown in Figure 1, the transfection efficiency of the M107 vector (eGFP) was 88.57% as compared to 56.58% for the vector containing LDLR–eGFP. However, after transduction of 293T cells with the same volume of eGFP or LDLR–eGFP supernatant, transduction efficiency for eGFP was 42.32%, compared to 1.55% in cells transduced with the LDLR–eGFP supernatant (Figure 1). It seems that the packaging of the cassette including the LDLR-eGFP was not successful. Furthermore, subsequent assays confirmed a negative effect of the transmembrane LDLR sequences on packaging of eGFP alone, indeed, co-transfection of hLDLR with eGFP and other packaging plasmids, led to the inhibition of eGFP packaging in a dose-dependent manner [35].

### 3.2. Docking Calculation and Protein–Protein Interaction Determination through MD Simulation

Because the inhibitory mechanism for the LDLR protein is unknown, we used bioinformatics methods to identify a protein interaction between LVV and LDLR. After performing docking calculations and protein–protein interaction MD simulations, we found that the interface of all domains and seven conserved residues in the active site of LDLR interact with the gag-conserved domain, and that the most energetically favorable site for binding was the region between aa 295 and 690 (Supplementary Figure S2C). The interactions between LDLR and gag protein occur through the establishment of two hydrogen bonds connecting GlnA665, AsnA667 (LDLR), and AsnB49 (gag-conserved domain) (Supplementary Figure S2D). This region in the LDLR is important because of the essential role it plays in conformational changes in LBD, which in turn may be closely related to a loss of binding (Supplementary Figure S2E). Subsequent protein predictions were performed under high-binding affinity conditions to determine whether conformational changes would occur or be constrained. We also replaced a short hydrophobic region in the hLDLR ligand-binding domain. This substitution allowed us to identify specific conserved amino acids that show high affinity for interacting with the hLDLR ligand.

### 3.3. Functional Effect of Mutations and Sequential Deletions at the 3′ End of LDLR cDNA

**Figure 2:**
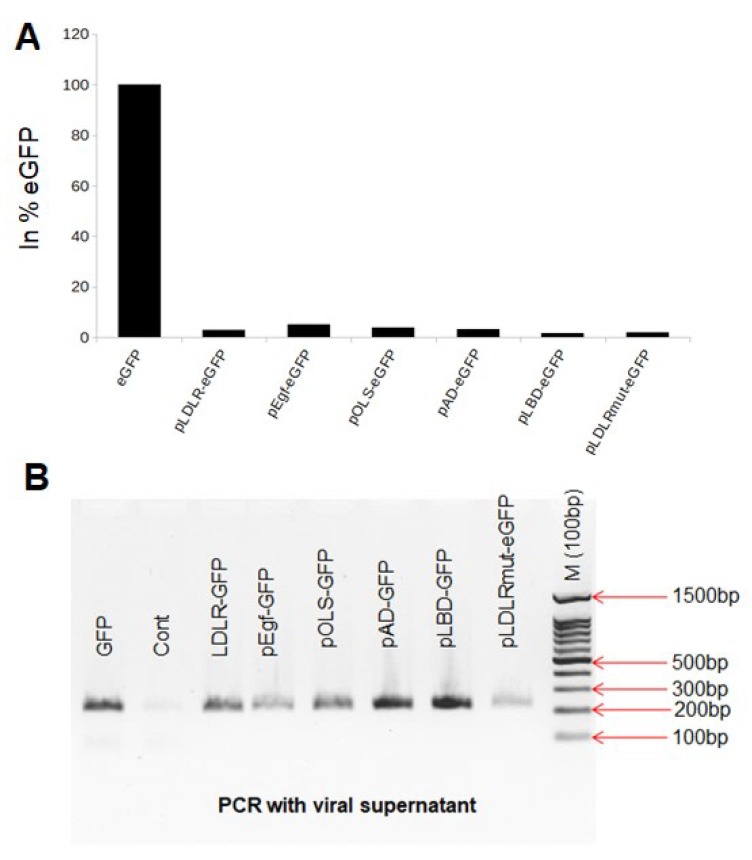
Transduction of 293T cells with LDLReGFP supernatant including different substitution or deletions at the 3’ of LDLRcDNA. The packaging of the construct harboring eGFP gene only or 5’-LDLReGFP-3’ cassette with different substitutions or deletion was done as described in the materials and methods section, thereafter we used the same supernatant amount to transduce the 293T cells. (**A**) We analyzed the expression of GFP using flow cytometry and express the transduction efficiency in % of GFP. (**B**) After isolation of RNA from the supernatant and synthesis of the first-strand cDNA, as described in the materials and methods section, the GFP fragment was showed using PCR.

To reduce the interaction between the gag protein and LDLR, we designed seven mutants. We also designed constructs with sequential deletions at the 3’ end of the LDLR cDNA in the 5′-LDLReGFP-3′ cassettes. After transfection and transduction, we quantified the transduction efficiency using FACS analysis. We found that transduction with supernatant containing LDLR–eGFP harboring seven mutations (pLDLRmut–eGFP) did not show any improvement compared to the LDLR–eGFP, with efficiency reaching 0.2% for LDLR–eGFP as compared to 0.13% for LDLRmut–eGFP (Figure 2A). Transductions with constructs containing sequential deletions at the 3′ end showed a positive signal in fewer cells as compared to cells with the LDLR–eGFP construct (Figure 2A). Further, RT-PCR analysis of the supernatant revealed that the pseudovirus from different constructs contained the eGFP sequence, indicating that successful packaging of viral RNA in the VSV envelope had occurred (Figure 2B).

### 3.4. Effect of a Liver-Specific Promoter and Sequential Deletions at the 5′ End of the LDLR cDNA on the Packaging of LDLR–eGFP

**Figure 3:**
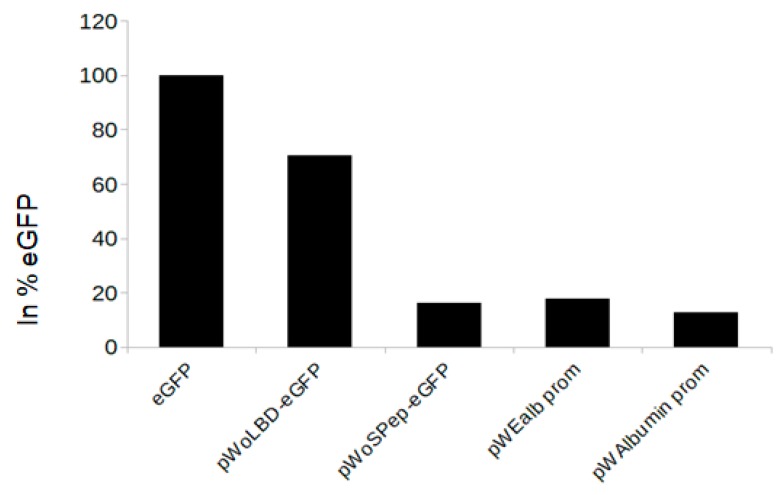
Transduction of HepG2/293T cells with supernatant from constructs under liver-specific promoter or presents deletions at 3’ of LDLReGFP. After transfection with constructs harboring eGFP gene only, constructs with LDLReGFP cassette under the liver-specific promoter or LDLReGFP constructs with deletions at 3’ of LDLReGFP, the same amount of the supernatants was used for the transduction of the HepG2/293T cells, as described in the materials and methods section, the transduction efficiency was presented in % of GFP.

Initially, we tested the interactions between the gag and LDLR, however the experimental assay showed that the seven modifications in the LDLR (pLDLRmut-eGFP), and the deletion at the 3’ end, do not improve the packaging. Then, based on the publication from Finkelshtein et al. [34], we designed additional constructs with deletions at the 5’ end and tested the interactions between the LBD and SVS-G. Furthermore, we also substituted the CMV promoter into an LDLR–eGFP construct containing a liver-specific promoter (albumin or Ealb). The transfection efficiency was comparable for all constructs (Supplementary Figure S3) and reached 54.96% for eGFP, 40.71% for the albumin promoter-containing construct (pWAlbumin prom), 54.73% for the Ealb promoter-containing construct (pWEalbprom), 46.44% for the construct without a signal peptide (pWoSPep-eGFP), and 41.77% for the construct without LBD (pWoLBD-eGFP). When 293T/HepG2 cells were transduced with the same volume of pseudovirus supernatant, we found that pWoSPep-eGFP, pWEalbprom, and pWAlbumin had low transduction efficiencies (Figure 3). In contrast, transduction with pWoLBD–eGFP-containing supernatant demonstrated an improved transduction efficiency of ~70% of the eGFP transduction (Figure 3).

### 3.5. Interaction between VSV-G Protein and LDLR

**Figure 4:**
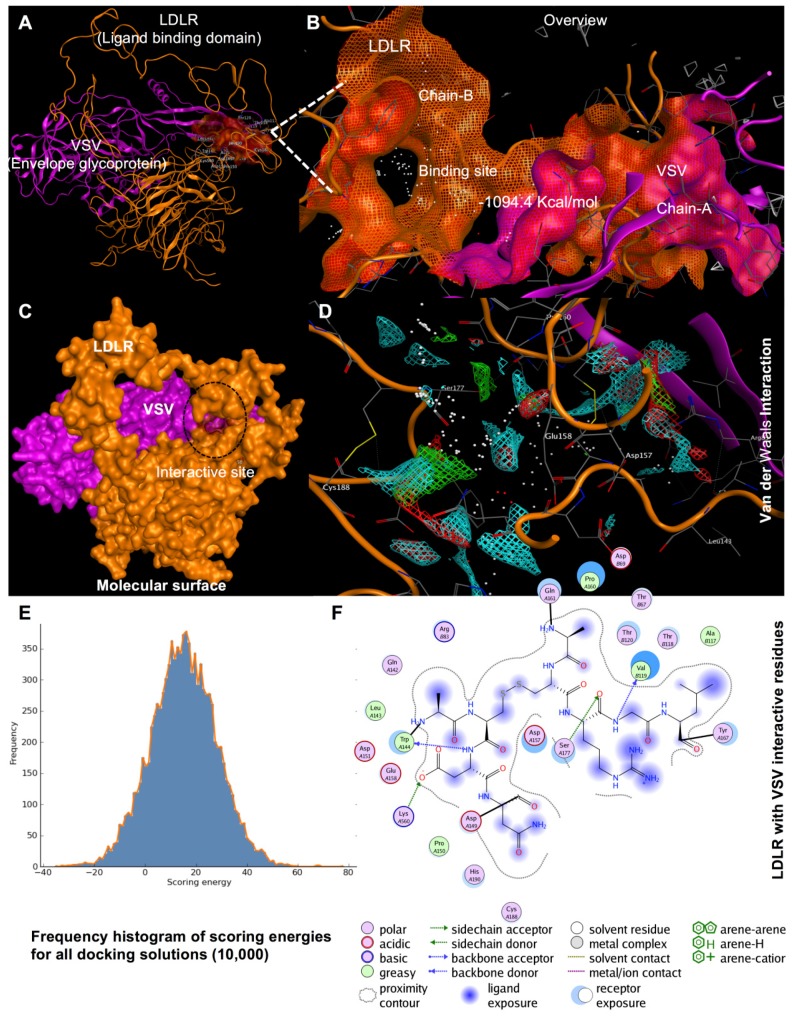
Model of the crystal structure of VSV and LBD interactions. Protein to protein interaction with LBD of LDLR (Chain **A**) PDB ID: 1n7d (orange) and VSV envelope glycoprotein g ecto domain (Chain **B**) ID: 5i2s (purple). (**B**) LBD active site as shown in the molecular surface. (**C**) LBD as shown in the molecular surface with a target VSV was highlighted in red. (**D**) A complex of LBD and VSV envelope glycoprotein has a van der Waals interaction with each other as shown in residues (atom) for binding are labeled with three-letter amino acid codes. Furthermore, an overview of LDLR ligand binding domain and VSV interact with each other as shown helix model. (**E**) Frequency histogram of docking scoring energies barrier for docking. (**F**) The interactive residues both VSV and LBD in n2D view. MOE was used to generate the figure based on the information.

To our knowledge, no studies are evaluating a direct interaction between LDLR and VSV envelope glycoprotein using MD simulation. Protein–protein interaction surfaces are generally hydrophobic. We assessed hydrophobicity by measuring the area of an accessible protein surface that forms an interface region with a partner protein, thus, becoming inaccessible to the solvent owing to protein–protein contact. In the simulation, the two proteins LDLR and VSV were treated as rigid objects, and 6-dimensional rotational and translational degrees of freedom were explored. For this phase, surface complementarity, electrostatic complementarity, and desolvation parameters were selected to probe various conformations by using a fast Fourier transform. Several confirmations were identified and ranked according to scoring criteria. We used the result of a distance restraints calculation, together with electrostatic and desolvation binding energy and affinity, to identify the correct docking orientation. Out of the top 10 highest energies for these protein–protein interactions, the lowest energy score was 1094.4 kcal/mol (Figure 4), corresponding to the proposed interaction between the LDLR LBD and VSV envelope glycoprotein. The exposed residues of the LBD in the LDLR receptor that interacted with the VSV protein were Val119, Thr120, Thr67, and Thr118. Besides, LDLR has a side chain donor containing residue Asp69 and a backbone acceptor containing residue Arg83 that strongly interact with each other.

## 4. Discussion

FH is a life-long autosomal codominant disease in which ~90% of patients have defects in the LDLR. To date, the treatment of FH, especially for homozygous individuals, is challenging despite efforts to manage the disease with lifestyle changes and/or use of drugs. The majority of patients with homozygous FH maintain high serum LDL-C levels [10] and are at high risk for CVD. Plasma apheresis or liver transplantation opportunities are limited, and thus, delivery of a functional LDLR transgene shows great promise as a therapy. Previously, retroviral [20,21] or adenoviral [46] vectors or AAV was used to deliver LDLR transgene [22,23]. In this study, we used an LVV pseudotyped with VSV envelope. This envelope has been extensively studied [47,48] and is characterized by broad tropism, high stability, and excellent transduction efficiency with multiple pseudotyped viral vectors [32,49,50]. 

We were able to achieve a transduction efficiency of 42.32% using supernatant containing eGFP, but the efficiency was only 1.55% for cells transduced with LDLR–eGFP supernatant. The low transduction efficiency in our study is comparable to that obtained by Grossman et al., where only a few transduced hepatocytes and a small reduction in LDL-C levels were observed in three subjects [21], despite promising results from studies in rabbits with LDLR [20]. In contrast, Ou et al. obtained a remarkable reduction in LDL levels [51], although transduction efficiency was not provided in the analysis of LDLR mRNA and protein expression levels. Interestingly, we also found that LDLR affects the packaging of eGFP alone. Use of an LVV pseudotyped with VSV envelope and co-transfection of hLDLR with the eGFP plasmid in addition to the other packaging plasmids led to inhibition of eGFP packaging in a dose-dependent manner [35]. This negative effect of LDLR on the packaging of eGFP was also confirmed in the publication from Otaha et al. [35]. However, we do not know the mechanisms by which the expression of the LDLR inhibits the packaging of the eGFP. It is not because of the length or structure of the transgene cassette, but it could be related to the protein–protein interactions between the components of the LVV system, including the LDLR and the VSV envelop protein.

To investigate reduced packaging efficiency, we designed, using bioinformatics methods, several LDLR mutants and generated constructs with sequential deletions at the 3′ and 5′ ends of the LDLR cDNA in the 5′-LDLReGFP-3′ cassettes. We also replaced the CMV promoter with liver-specific albumin and Ealb-Pa1AT promoters. However, these mutations did not improve packaging efficiency (Figure 2A), and only a few positive cells were observed compared to positive signal observed for the eGFP construct, despite verification of successful viral RNA packaging in the VSV envelope (Figure 2B). Also, neither deletion of the signal peptide nor substitution of the CMV promoter with liver-specific promoters increased the transduction efficiency (Figure 3). In contrast, removing the LBD of LDLR (pWoLBD-eGFP) allowed transduction efficiency to reach approximately 70% of the eGFP transduction (Figure 3), which led us to conclude that this domain is responsible for low packaging efficiency. 

Our result is consistent with that of the Rubinstein group [33,34], who found that interferons induce an extracellular soluble protein, identified as soluble LDLR (sLDLR), that inhibits VSV infection [33]. Recently, this group also analyzed the effect of sLDLR on eGFP expression after transduction of cells with eGFP packaged in an LVV pseudotyped with VSV-G and showed that sLDLR completely blocked transduction of fibroblasts. In contrast, sLDLR did not inhibit transduction of cells with eGFP packaged in an LVV pseudotyped with lymphocytic choriomeningitis virus (LCMV), indicating that sLDLR specifically binds to VSV-G and that the sLDLR must be present at the early stages of viral infection to exert its antiviral effects [34]. Using co-immunoprecipitation, the authors also found that interactions exist between the ligand-binding domain of LDLR and VSV-G. Which is consistent with our results and showed that removing the LBD increased the transduction efficiency. Furthermore, the MD simulation showed that the exposed residues of the LDLR receptor were Val119, Thr120, Thr67, and Thr118, in the LBD that interacts with VSV. As LBD is the most important region in the transport of LDL, it is difficult to modify it without affecting the transport efficiency of LDLR. For this reason, in future studies, it would be more suitable to replace the VSV envelop with the LCMV envelope, or to use the murine leukemia virus (MLV) pseudotyped with the hepatitis B virus (HBV) envelop, to obtain higher transduction efficiencies. Previous studies have shown the possibility of pseudotype LCMV glycoproteins with retroviral and HIV-based lentiviral vectors for gene transfer [52,53]. Moreover, the group of Dylla et al. used feline immunodeficiency virus (FIV) vectors pseudotyped with the LCMV-derived glycoproteins for successful transduction in the liver [53]. For the murine leukemia virus (MLV) or the LVV pseudotyped with the hepatitis B virus (HBV) envelop, previous studies approved the effectivity of this system for gene transfer in primary human hepatocytes [54,55].

## 5. Conclusions

Taken together, our results show that the LBD of the LDLR interacts with the VSV-G protein during the packaging process in a lentiviral VSV-G system, and this interaction significantly reduces transduction efficiency. While it is possible to use an LVV system for packaging the LDLR–eGFP construct, another envelope from other viruses, such as LCMV or MLV, should be used to avoid this reduced efficiency.

## Supplementary Materials

Supplementary materials can be found at https://www.mdpi.com/1999-4915/11/11/1063/s1.
