# Supplementary figures and images for: Molecular Dynamics Simulation Reveals Exposed Residues in the Ligand-Binding Domain of the Low-Density Lipoprotein Receptor that Interacts with Vesicular Stomatitis Virus-G Envelope
Source: Viruses. 2019 Nov 15;11(11):1063. doi: 10.3390/v11111063 (PMC6893590; doi:10.3390/v11111063)

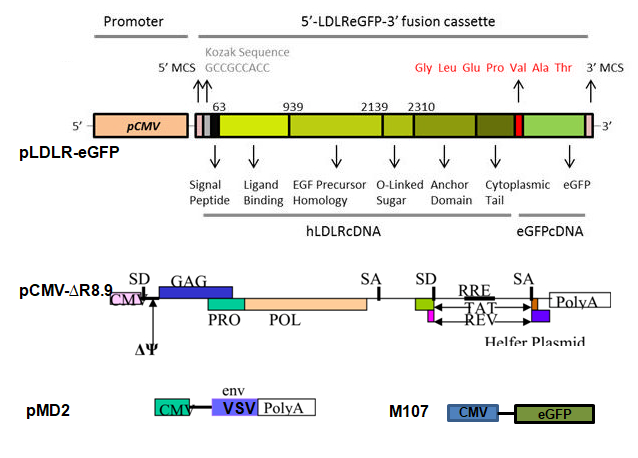

Supplement: Supplementary file 1 [file viruses-11-01063-s001.zip › New folder/Sup Figure 1.tif]

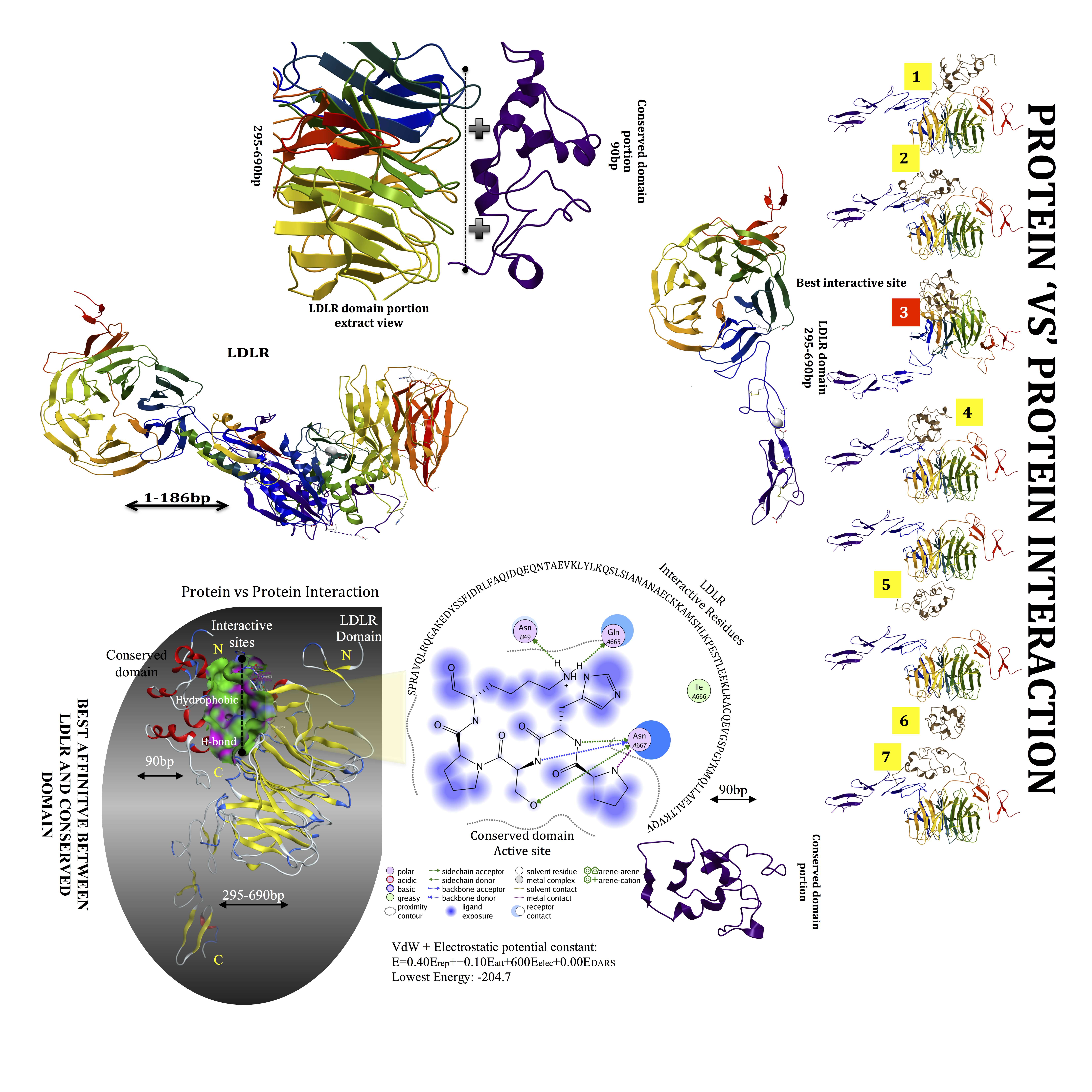

Supplement: Supplementary file 1 [file viruses-11-01063-s001.zip › New folder/Sup Figure 2.tiff]

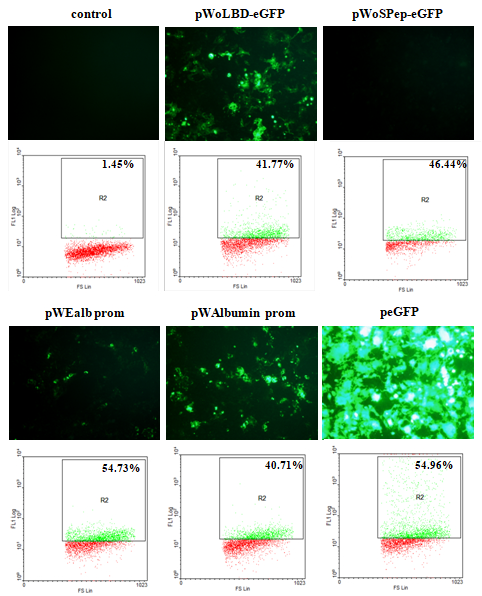

Supplement: Supplementary file 1 [file viruses-11-01063-s001.zip › New folder/Sup Figure 3.tif]

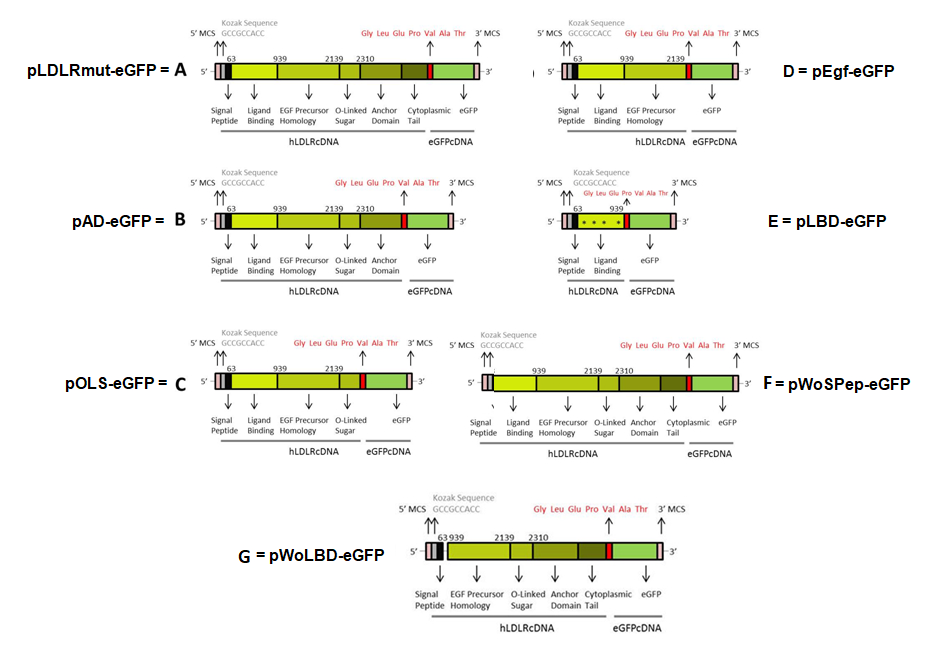

Supplement: Supplementary file 1 [file viruses-11-01063-s001.zip › New folder/Sup Figure 4.tif]
